# Novel, Innovative Models to Study Ischemia/Reperfusion-Related Redox Damage in Organ Transplantation

**DOI:** 10.3390/antiox12010031

**Published:** 2022-12-24

**Authors:** Julia Hofmann, Marlene Pühringer, Sabrina Steinkellner, Aline-Sophie Holl, Andras T. Meszaros, Stefan Schneeberger, Jakob Troppmair, Theresa Hautz

**Affiliations:** OrganLife Laboratory and Daniel Swarovski Research Laboratory, Department of Visceral, Transplant and Thoracic Surgery, Center of Operative Medicine, Medical University of Innsbruck, 6020 Innsbruck, Austria

**Keywords:** Ischemia-reperfusion injury, redox-stress, organ transplantation, machine perfusion, in vitro models

## Abstract

The implementation of ex vivo organ machine perfusion (MP) into clinical routine undoubtedly helped to increase the donor pool. It enables not just organ assessment, but potentially regeneration and treatment of marginal organs in the future. During organ procurement, redox-stress triggered ischemia-reperfusion injury (IRI) is inevitable, which in addition to pre-existing damage negatively affects such organs. Ex vivo MP enables to study IRI-associated tissue damage and its underlying mechanisms in a near to physiological setting. However, research using whole organs is limited and associated with high costs. Here, in vitro models well suited for early stage research or for studying particular disease mechanisms come into play. While cell lines convince with simplicity, they do not exert all organ-specific functions. Tissue slice cultures retain the three-dimensional anatomical architecture and cells remain within their naïve tissue-matrix configuration. Organoids may provide an even closer modelling of physiologic organ function and spatial orientation. In this review, we discuss the role of oxidative stress during ex vivo MP and the suitability of currently available in vitro models to further study the underlying mechanisms and to pretest potential treatment strategies.

## 1. Introduction

Organ transplantation remains the ultimate treatment option for terminal organ failure. However, the number of organs in demand surpasses the number of available organs leading to a significant organ shortage [1]. Owed to the implementation of advanced preservation technologies (i.e., machine perfusion), increased-risk organs from extended criteria donors (ECD) can be considered for transplantation [2,3,4,5]. However, these organs are particularly prone to additional damage during organ retrieval, preservation and transplantation. In this regard, ischemia-reperfusion injury (IRI) caused by oxidative stress and subsequent events during early reperfusion negatively affects the short- and long-term outcome after transplantation, since several molecular downstream pathways are activated, further aggravating pre-existing damage [6,7]. It could be demonstrated that machine perfusion (MP) may mitigate oxidative stress mediated injury; however, the underlying mechanisms are not fully understood at this point [8,9,10]. 

In addition to whole organ perfusion experiments, which are expensive, complex and sophisticated, in vitro models such as cell lines, precision-cut tissue slices (PCTS) and organoids may be helpful, when addressing specific research questions [11]. Moreover, awareness about using animals in research has increased over the past decades. Besides economic reasons, using in vitro models can eliminate animal experiments in compliance with the 3R (replacement, reduction, refinement) principles [12]. 

In this review, we summarize the key mechanisms of oxidative organ damage with a particular focus on ex vivo organ perfusion, highlight possible treatment strategies and provide an insight into suitable in vitro models complementary to the in vivo perfusion setting.

## 2. IRI Is the Key Event Leading to Oxidative Stress in Organ Transplantation

**Figure 1:**
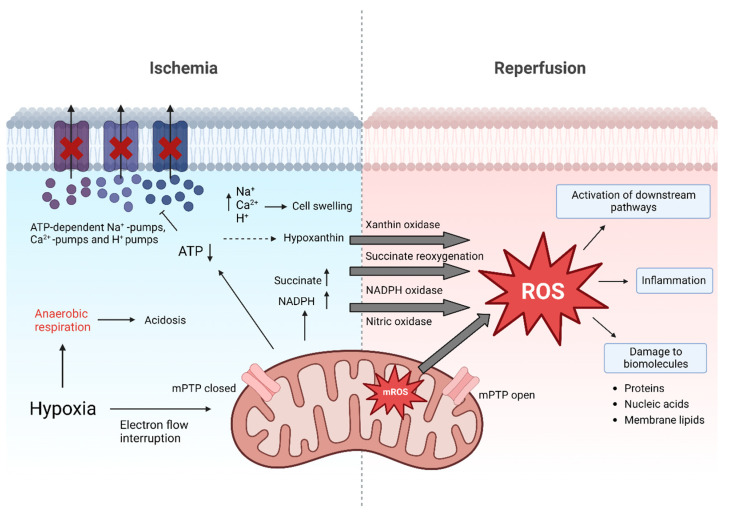
Molecular events of ischemia and reperfusion. During ischemia, adenosine triphosphate (ATP) levels decrease. In turn, ATP-dependent Ca2+, H+ and Na+ pumps fail, causing accumulation of ions which contributes to cell swelling. pH levels decrease leading to acidosis. Accumulation of succinate, nicotinamide adenine dinucleotide phosphate (NADPH; resulting from NADP+ and H+) and hypoxanthine during ischemia prime for excessive ROS release after reperfusion. Additionally, in mitochondria major reactive oxygen species (ROS) generation occurs. ROS cause direct damage to biomolecules but also act as signaling molecule. Besides this, opening of the mitochondrial permeability transition pore (mPTP) during reperfusion also triggers cell death by release of cytochrome c and breakdown of ATP production [5,16].

Broadly, the imbalance between reactive oxygen species (ROS) generated and antioxidants present is known as oxidative stress [13]. In the setting of solid organ transplantation, one of the most common ROS-related pathologies is IRI [7,11]. IRI is inherently connected to organ transplantation. It is characterized by obstructed blood flow causing ischemia during organ retrieval and preservation, followed by a reperfusion phase when the blood flow is restored in the recipient [10,14,15,16] (Figure 1).

The organ retrieval process kicks off a cascade of molecular events that eventually set the basis for ROS release. The interrupted oxygen supply inhibits the mitochondrial electron transport chain, resulting in a decreased production of adenosine triphosphate (ATP) [6,17,18,19]. The subsequent shift to anaerobic metabolism leads to the retention of hydrogen (H+) ions and retained metabolic products such as lactic acid, resulting in metabolic acidosis. This decreases the cellular pH, which further leads to clumping of chromatin and impaired enzyme activity. Moreover, the ATP-dependent sodium-potassium, calcium and sodium-hydrogen pumps fail during ischemia, resulting in increased intracellular H+, sodium (Na+) and calcium (Ca2+) concentrations, which cause swelling of the cells. The partial reversal of the malate-aspartate shuttle and degradation of purine nucleotide results in an excess of fumarate, leading to a reversal of succinate dehydrogenase (SDH), ultimately causing succinate accumulation [20]. During early reperfusion it is rapidly degraded and through complex metabolic pathways it contributes to a burst in ROS production at complex I of the ETC. Additionally, nicotinamide adenine dinucleotide phosphate (NADPH) oxidases, xanthine oxidase and nitric oxidase synthase are also involved to increase ROS production under these conditions. Thus, while necessary for prolonged organ survival, the reperfusion phase in the recipient exacerbates cellular injury, which already occurred during ischemia [19,21,22,23]. Reperfusion injury is a progressive condition post-transplantation, that can last for multiple days, negatively affecting early graft function as well as long-term graft survival [19]. 

Free ROS can cause direct damage to biomolecules by oxidation of proteins, oxidation of nucleic acids or peroxidation of membrane lipids, ultimately resulting in cell death [6]. On the other hand, ROS are also known for their function as signaling molecules. Signaling proteins can be phosphorylated and thereby activated by ROS. Mitochondrial-activated protein kinases (MAPK), namely extracellular signal-regulated kinase (ERK1/2), c-Jun N-terminal kinase (JNK), and p38, play an important role in this cascade [24]. Phosphorylation and activation of ERK1/2 has been associated with neutrophil infiltration, necrosis, and apoptosis in rodent models of liver IRI [25,26]. The phosphorylation of JNK can lead to an increase and activation of apoptosis-promoting molecules such as Bim, Bad, Bax, and p53 [27,28,29,30]. On the other hand, phosphorylation of JNK also causes downregulation of survival signals involving STAT3 [28,30]. After reperfusion, phosphorylation and hence activation of p38 is initiated, which is directly related to apoptosis and necrosis. Its activation leads to an increase in adiponectin, which in turn enhances ROS release, resulting in tissue damage [6]. Further, apoptosis is induced by the release of cytochrome c, which in turn activates caspase-9, resulting in caspase-3 induced apoptosis. Moreover, a cascade of proinflammatory signaling pathways is induced by oxidative stress including the generation of inflammasomes [6]. Consequently, different pro-inflammatory cytokines are secreted, whereas TNFα is considered as a decisive factor for further triggering the downstream inflammatory cascade. In addition to this ‘humoral answer’ of the immune system, also the innate immune system is activated by the release of damage-associated molecular patterns (DAMPs), triggering the activation of dendritic cells (DCs), macrophages and natural killer cells (NKs) [31]. These molecular processes in combination with the lower concentration of antioxidative agents, ATP depletion and calcium dysregulation are considered the main drivers of IRI [20]. However, ROS release is not harmful by definition. This dose response phenomenon characterized by a low dose stimulation and a high dose inhibition has been observed in response to many exogenous stimuli and is referred to as hormesis in the literature [32,33]. There is growing evidence, that the lack of necessary ROS is detrimental. Together with the accumulation of reductive equivalents during ischemia, the absence of ROS is responsible for reductive stress. In this regard, three of the major couples of the cellular redox network are NAD+/NADH, NADP+/NADPH and GSH/GSSG. Like oxidative stress, also reductive stress contributes to the overall redox stress resulting in impaired cellular functions [34,35].

### 2.1. Molecular Mechanisms Counteracting Oxidative Stress 

In order to counteract ROS, organisms exhibit their own enzymatic and non-enzymatic antioxidant defense systems. Since they may not be sufficient for averting oxidative stress, several regulatory pathways to counter it exist. They may serve as targets for treatments, which will be discussed further below [36]. One of the most important transcription factors in this regard is the Keap1-Nrf2 pathway [37]. Nuclear factor-erythroid-2 related factor (Nrf2) is a ubiquitously expressed transcription factor regulated by the repressor protein Kelch-like ECH associated protein1 (Keap1). Upon oxidative stress, Keap1 dissociates from Nrf2, and Nrf2 can subsequently enter the nucleus and attaches to antioxidative response element (ARE). In turn the transcription of antioxidative enzymes like superoxide dismutase (SOD) or glutathione peroxidase (GPx) is induced [37]. Moreover, mammalian target of rapamycin (mTOR), which is part of the phosphatidylinositol 3-kinase/protein kinase B (PI3K/Akt) pathway, as well as forkhead transcription factor O (FOX) have also been described as part of the antioxidant regulatory system [38,39]. Additionally, proteins regulated by the transcription factor nuclear factor-κB (NFκB) can be activated upon degradation of inhibitor of κB (IκB) to regulate the amount of ROS in the cell [40]. However, in the context of IRI, the role of NFκB is quite controversial. For instance, activation of NFκB in the liver has been shown to reduce hepatic IRI injury and facilitate orthotopic liver transplantation [41], while another group observed protection against hepatic IRI injury upon inactivation of NFκB [42]. 

Despite technical and therapeutical improvements, oxidative stress-induced IRI remains an inevitable event and still, mechanisms triggered by ROS release are not fully decrypted. Thus, potential models which help deciphering mechanisms and allow for testing of therapeutical approaches will be discussed in Section 3 and Section 4.

### 2.2. Biomarkers to Study Oxidative Stress

As direct contributors to oxidative stress, ROS should be considered as potential biomarkers [6,21]. Direct detection would allow for quantification of oxidative stress. However, due to the short half-life of ROS, this is currently a very complex method [43]. Instead of tracking ROS itself, their effects on biomolecules can be detected. Alterations in expression or formation induced by ROS can be used as valuable surrogate biomarkers for oxidative stress. Roughly, these molecules can be categorized as follows: endogenous antioxidants, lipid peroxidation, oxidative protein changes and nucleic acid oxidation [44]. Representative examples are listed in Table 1 and discussed in the following sections.

These studies undermine distinct patterns in the antioxidative profile between perfused and SCS grafts. Complementary to the analysis of those surrogate parameters, analysis at protein and gene expression levels to evaluate changes in pathway activation might provide a more comprehensive profile [58]. Moreover, quantification of known cytokines to be involved in oxidative stress response should be considered.

#### 2.2.1. Endogenous Antioxidants

Organisms possess defense systems against free radicals, one being facilitated by antioxidant enzymes [13]. These can be quantified and serve as biomarkers. Catalase (CAT) is an enzyme found in almost all living organisms that are exposed to oxygen. Within the field of transplantation, it is most widely used to assess oxidative stress [59]. Another antioxidant is SOD, an enzyme group that acts as a crucial part of the antioxidant defense against highly reactive superoxide radicals. It is responsible for splitting (dismutation) of H_2_O_2_ [60]. GPx can reduce H_2_O_2_ or organic peroxides to water and alcohol with the presence of glutathione and is subsequently converted to oxidized glutathione [44].

#### 2.2.2. Lipid Peroxidation

It is known that ROS can promote the formation of fatty acid radicals [58]. These unstable fatty acid radicals can subsequently react with molecular oxygen to form peroxides. Moreover, lipid peroxidation products can lead to the synthesis of, for instance, malondialdehyde (MDA) [61]. MDA and the reactive thiobarbituric acid substance (TBARS) are considered basic markers of lipid peroxidation, potentially serving as biomarkers [62]. Additionally, isoprotanes serve as valuable markers, where F2 and F4 isoprotanes should be distinguished. F2 isoprotanes are formed by free radical catalyzed peroxidation of arachidonic acid, whereas F4 is a product of the same reaction of docosahexaenoic acid. It is also interesting to note that F4 isoprotanes exert a strong anti-inflammatory effect, which underlines the link between oxidative stress and inflammation [63,64].

#### 2.2.3. Redox Modification of Proteins

When it comes to protein changes due to oxidative stress, 3-nitrotyrosin is considered as one of the most promising biomarkers [44]. Nitration of protein-bound and free tyrosine by ROS leads to the formation of this molecule. Besides nitrotyrosines, protein carbonyls are also widely used as biomarkers for oxidative stress [65,66].

#### 2.2.4. Nucleic Acid Oxidation

Oxidative DNA damage, mostly caused by the hydroxyl radical, generates a variety of base and sugar modification products [6,67]. DNA damage caused by hydroxyl radicals occurs much less frequently than oxidative protein changes. However, the consequences of nucleic acid oxidation, such as mutations, are considerably more harmful. Although hydroxyl radicals can react with all purine and pyrimidine bases as well as with the deoxyribose backbone, the major products of oxidative nucleic acid changes are 8-oxoguanine and 8-hydroxy-2′deoxyguanosine [68]. 

Detection of those biomarkers can be performed in tissue samples and plasma, serum or perfusate. The selection of suitable biomarkers is depending on the study and may not rely on a single analysis method rather than on supplementary methods. Physiological levels of antioxidative enzymes like SOD and GPx and their increase in response to oxidative stress may be a more sensitive method. In contrast, evaluation of damage to biomolecules require excessive oxidative stress and may be only detected in more severe forms of oxidative stress induced damage. 

## 3. Advanced Organ Preservation: Ex Vivo Machine Perfusion

**Figure 2:**
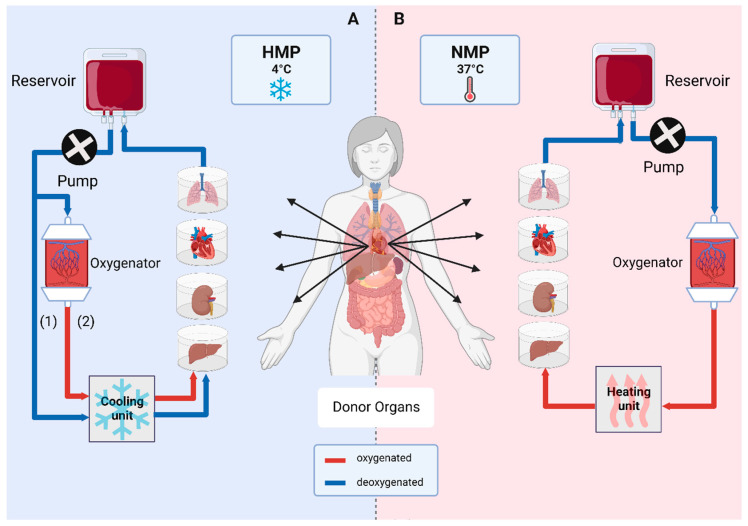
Ex vivo organ machine perfusion. Main vessels of an organ are cannulated and constantly perfused. (**A**) Hypothermic machine perfusion (HMP) is performed at 4 °C and can either take place without oxygenation (1) or with incorporation of an oxygenator (2), known as hypothermic oxygenated machine perfusion (HOPE). (**B**) Normothermic machine perfusion (NMP) is performed at 37 °C with oxygenated perfusion solutions under close-to-physiological conditions [3].

Throughout several decades, the gold standard for organ preservation has been static cold storage (SCS) at 4 °C [69]. Cellular metabolism and oxygen consumption are reduced at hypothermia, widely preventing damage to the tissue. During the retrieval process, organs are flushed with cold preservation solution in order to deprive the organ of blood, while providing cytoprotection. University of Wisconsin (UW®) Cold Storage Solution and Custodiol® histidine-tryptophan-ketoglutarate (HTK) Solution are most widely used for cold organ preservation, storage and transport. UW® solution contains glutathione, allopurinol and adenosine as antioxidative component, while HTK solution utilizes mannitol, tryptophan and α-ketoglutarate [2]. In the recent years, dynamic preservation by machine perfusion has found its way into clinics, which helped to increase the donor pool for abdominal and thoracic organs. This is of specific interest for, but not limited to, ECDs. Such marginal organs are often not considered for transplantation otherwise and predicting outcome remains difficult. Moreover, logistics and recipient-related issues are convincing reasons to opt for MP [4,70]. It has been proven that MP technologies are aiding in tackling problems like IRI and downstream inflammatory processes and improving graft function early after transplantation as well as long-term survival [71]. Different MP strategies operating at various temperatures are available and explored to different degrees (Figure 2).

### 3.1. Hypothermic Machine Perfusion (HMP)

Similar to SCS, HMP is carried out at 4 °C. Metabolism is reduced significantly to about 10%, which decreases energy demand and preserves ATP. Despite residual cellular function being left, oxygen supply is not routinely used in standard care. However, it could be demonstrated that the addition of oxygen carriers and providing oxygen supply to perform the so called hypothermic oxygenated machine perfusion (HOPE) exerts further beneficial effects. Superior outcome of HMP treated organs over SCS organs could be demonstrated in the past [3,71,72].

### 3.2. Subnormothermic Machine Perfusion (SNMP)

SNMP settles in between HMP and normothermic machine perfusion (NMP). Perfusion solutions rely on the physically dissolved oxygen at temperatures between 20–25 °C. Compared to HMP, partial testing of viability is possible during SNMP. However, it is not widely used and requires more research to assess feasibility [73].

### 3.3. Normothermic Machine Perfusion (NMP)

NMP is performed at 37 °C to mimic physiological conditions. Aerobic metabolism is restored in this MP modulation, therefore shortening ischemic time. Moreover, NMP enables organ assessment at a regular metabolic rate and offers the opportunity for treatment and direct manipulation of a graft prior to transplantation [74]. In order to supply the organ optimally, oxygenated perfusion solutions are necessary. These solutions can either be blood-based or acellular, containing hemoglobin-based oxygen carriers. So far, there is no clear evidence on what option to prefer- however, blood-based perfusates are the method of choice in most applications. Different protocols are used among the different research groups, however some common supplements in perfusate protocols are antibiotics, heparin, bicarbonate, vitamins and prostaglandins [75].

### 3.4. Influence of Perfusion Modalities on Oxidative Stress-Induced Tissue Damage

With regard to MP modalities and protocols, temperature, oxygenation and perfusate composition are the parameters of interest to adjust [76]. Lower temperatures decrease the mitochondrial oxygen consumption as well as the activity of other enzyme systems, which may result in a short-term beneficial effect of HMP [77,78]. In a study by Schlegel et al. it was demonstrated, that in HMP preserved DCD livers the mitochondrial redox state is altered, leading to decreased initial ROS release during reperfusion. Following that, less nuclear cell injury has been observed [8]. In HMP preserved kidneys the extent of oxidative stress was significantly reduced compared to the SCS group, indicated by significantly lower levels of glutathione peroxidase and malondialdehyde and superior graft function after transplantation [45]. In line with this, Venema et al. found reduced thiobarbituric acid-reactive substances (TBARS) in their study for HMP preserved kidneys [9]. However, Hendriks et al. report on higher oxidative stress levels at cold temperatures and decreased ROS scavenging capacity compared to 37 °C in a kidney perfusion model [79]. Even at 4 °C, the metabolic rate remains at around 10%, thus oxygenation during hypothermic preservation should be considered. In a multicenter clinical trial, superiority of HOPE in contrast to HMP was demonstrated for deceased kidneys [72]. On the other hand, NMP enables restoration of metabolic activity during preservation and therefore is the MP type of choice for functionality testing [2,77]. Falk et al. report on decreased IRI induced damage in human hearts preserved under normothermic conditions, indicated by decreased IRI-related inflammatory cytokines [80]. Moreover, technical feasibility to perform IRI-free organ transplantation by incorporating NMP devices could be demonstrated for the liver [81] and the kidney [82]. However, due to highly complex logistics and extremely high demand of human resources, this has not yet found its way into clinical routine.

### 3.5. Further Extension of the Donor Pool: Targeting Pre-Existing and Preservation-Induced Damage

Treatment of pre-existing morbidities and IRI induced damage during long-term perfusion may help to overcome organ shortage. NMP is the perfusion modality of choice, due to the restored metabolic activity during preservation [83,84,85]. Treatment approaches therefore require MP protocols that enable a sufficiently long therapeutic window. The Zurich group achieved a 7-day-NMP of discarded human livers without subsequent transplantation [86] and recently reported on a 3-day-NMP followed by successful transplantation [87]. Human graft ex vivo lung perfusion (EVLP) with subsequent transplantation was reported successful for three days in a pig model [88]. Most recently, NMP of human kidneys was shown feasible for 48 h [89], while in contrast heart MP is only possible for a few hours [90].

In line with preservation solutions utilized for SCS, perfusate composition and supplementation with protective agents like antioxidants can be considered to avoid and ameliorate oxidative stress during ex vivo organ perfusion. Antioxidants have the potential to scavenge ROS and thereby dampen oxidative stress. Some commonly used organ preservation solutions are supplemented with antioxidants for that reason. For example, allopurinol and glutathione are responsible for the antioxidant activity in Institute George Lopez-1 (IGL-1) preservation solutions [2,3]. The newer version of IGL-1, IGL-2, exhibits an even bigger antioxidant capacity by containing 3-fold increased glutathione levels. IGL-2 was utilized in a recent rat liver HOPE study where the authors could demonstrate superiority of IGL-2 vs. UW as perfusion solution. Hepatic endothelial glycocalyx were preserved and levels of caspase-3 and High mobility group protein B1 (HMGB1) were significantly reduced, underlining a protective effect of IGL-2 [91]. The group of Ehrsam et al. supplemented the perfusate for their rat lung perfusion with 2000 µM of β-nicotinamide adenine dinucleotide (NAD+), a coenzyme which is involved in removal of ROS and found reduced levels of pro-inflammatory cytokines and enhanced levels of anti-inflammatory cytokines [92]. In contrast, the addition of ascorbic acid during porcine kidney NMP did not significantly reduce oxidative stress [93]. Moreover, a variety of different agents with antioxidant properties may be suitable for application during MP to exert a beneficial effect on organ function and cell viability. These substances have been reviewed in detail recently elsewhere [94].

Another possible strategy to counteract IRI might be pharmacological inhibition of complex I prior and during MP to prevent ROS production by blocking this exact mechanism. Potential inhibitors are rotenone, metformin and small-molecule inhibitors [95,96]. Moreover, different groups report that targeting of the SDH dependent mechanism exhibits protective effects in IRI [97,98,99,100] and blocking of p38 has been shown to reduce IRI-induced necrosis and apoptosis [101,102,103].

## 4. Studying Oxidative Stress

**Figure 3:**
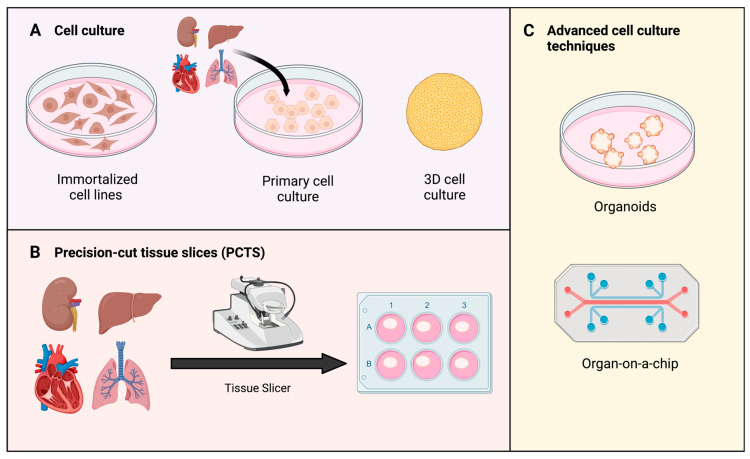
Types of in vitro models to study oxidative stress. (**A**) Cell culture can be sourced from immortalized and primary cell lines. In complex models, 3D cell cultures are prioritized over 2D monolayer culture [108]. (**B**) Precision-cut tissue slices (PCTS) are obtained from tissue of interest using a specific tissue slicer. Produced slices can be transferred and assessed in regular cell culture environment thereafter [109]. (**C**) Examples of advanced cell culture techniques: organoids and organs-on-a-chip. Organoids are 3D tissue structures grown from stem cells [110]. Organ-on-a-chip represent a new model to study impact of temperature, shear-stress, and oxygenation [111].

Even though MP was adopted in the clinical routine some time ago, valuable small-scale models are needed to study isolated processes in more detail. While they are not comparable, they are not mutually exclusive. 2D and 3D models can be used to study molecular mechanisms during IRI to further understand which interventions during MP are required or helpful to protect an organ (Figure 3). In order to induce the ischemic state, hypoxia together with acidosis, ATP depletion and accumulation of waste products are required [104,105]. Next, normoxic conditions and supply of nutrients are restored to allow for ROS generation- resembling the reperfusion state. In regard to IRI, shear stress induced by blood flow should also be taken into account in in vitro systems [105]. Connecting conventional cell culture plates to peristaltic or pulsatile pumps and thereby generating flow, has been described [106,107]. Following models discussed below focus on in vitro models that can be translated to MP research to test hypotheses in a more complex approach.

### 4.1. Cell Lines

In general, cell culture models are widely used to study different research questions. Most importantly, immortalized and primary cell lines are to be distinguished (Figure 3A). Immortalized cell lines are able to proliferate indefinitely, as they are mostly obtained from tumors. However, also methods for the immortalization of primary cells have been established [108]. Cell isolation involving enzymatic or mechanical disruption and further cultivation are frequently accompanied with the loss of some cell intrinsic features. In contrast, primary cells are directly isolated from tissue. However, they exhibit biological variability and can only be used for a limited duration due to a shorter life-span and cultivation-induced changes. In addition to typical 2D cell culture models, 3D techniques allow cells to grow into so-called spheroids. Cell–cell adhesion features are retained in those [106,108,111].

For most pathologies, a suitable cell line model is available nowadays. Induction of IRI has been successfully reported in cell lines of kidney, liver, heart and lung [112,113,114,115]. Módis et al. used the immortalized HepG2 liver cell line to study the cryoprotective effects of adenosine and inosine on liver IRI. The cells were first pretreated with adenosine and inosine, before glucose and oxygen deprivation were induced by medium change and alteration of the atmosphere in the incubator (to 95% N_2_:5% CO_2_ mixture). The following re-oxygenation phase was prompted by restoring normal culture conditions (5% CO_2_ atmosphere, 20% O_2_). Cell viability and cell cytotoxicity assays both showed, that adenosine and inosine exert cryoprotective effects [112]. Cell lines have been also widely used to study targeting of the protective mechanisms to counteract IRI. Several studies have been performed using a hypoxia/reoxygenation model of cells to investigate the Nrf2 pathway [116,117,118]. Ge et al. examined the relationship between Brahma-related gene-1 (Brg1), Nrf2/HO-1 signaling, and IRI. They showed that restoration of Brg1 during reperfusion can enhance Nrf2-mediated inducible expression of HO-1 during hepatic ischemia reperfusion. This resulted in increased antioxidant capacity to combat hepatocyte injury [116]. Using a different hypoxia/reoxygenation model, the protective effect of sevoflurane against hepatic IRI was demonstrated through regulation of the Nrf2/HO-1 pathway [117]. Moreover, the protective effect of activation of the PI3/AKT pathway in IRI has been repeatedly investigated using hypoxia/reoxygenation HK2 and H9c2 cell lines [119,120,121].

However, models representing IRI in the context of extracorporeal (re-) perfusion and MP require more than control of O_2_ levels. Mechanistic impacts of changes in flow and oxygenation parameters during retrieval and transplantation are of great importance and so is perfusate composition. In vitro models that take into account vessel type, size, pressure and size of the animal were developed to represent physiologic shear stress values. Depending on hypothesis, laminar, pulsatile laminar or perturbated flow are applied [122,123,124,125,126]. Despite the disadvantage of cell lines not recapitulating the complexity of solid tissue, they allow for easy and high-throughput testing of different drugs. Therefore, cell lines might be not the model of choice to study complex whole-organ mechanisms, but potentially serve as a model to test oxidative stress treatment strategies which can be applied during ex vivo machine.

### 4.2. Precision-Cut Tissue Slices (PCTS)

As viable ex vivo explants of the studied organ, precision-cut tissue slices (PCTS) offer preservation of the complex anatomical architecture with all different cell types in their native environment. As a result, original intracellular, cell–cell and cell-matrix interactions remain intact, which is a major advantage over conventional in vitro models [109,127]. The original production of slices with hand-held blades has evolved significantly to the point where reproducible and comparable slices can be generated with a thickness of 200–300 µm [128]. In 1980, Krumdieck and colleagues presented a device for producing tissue slices that were thin enough for all cell layers to be sufficiently supplied with oxygen and nutrients. Therefore, they were considered mini-models of the organ under study. Notably, they can be prepared of liver, kidney, heart and lung tissue [129,130,131,132]. In 2010, Graaf et al. published a protocol, which still serves as gold standard to date [109]. Once PCTS are transferred into cell culture, their handling is as simple as culturing cells. Conventional cell culture plates can be utilized. Ideally, the incubator should be equipped with a shaking platform, in order to improve oxygen and nutrient distribution. Viability and metabolic activity of are mostly monitored by investigating ATP content and assessment of the NADPH-dependent oxidoreductase enzymes by 3-(4,5-dimethylthiazol-2-yl)-5-(3-carboxymethoxyphenyl)-2-(4-sulfophenyl)-2H-tetrazolium (MTS) assay, but not limited to these. Cell damage may be evaluated by analysis of lactate-dehyrogenase (LDH), aspartate aminotransferase (AST) and alanine transaminase (ALT) in the supernatant. In general, analysis of PCTS supernatant can be conducted analogous to MP supernatant [127].

Nowadays, PCTS are not only a useful tool for biochemical functions and toxicological studies, but also to study physiology and pathogenesis. Quick and reproducible results, as well as the presence of spatial heterogenicity with nutrient and oxygen gradients make PCTS a very sophisticated yet feasible model approach. In contrast, when using discarded human organs with a broad variety of pre-existing tissue damage and/or diseases, the heterogeneity of tissue slices is a major limiting factor. Healthy human tissue is most often only sporadically available, which complicates experiments and makes it difficult to obtain comparable, standardized cohorts. Furthermore, studying chronic and long-term effects is not possible to date due to limited cultivation periods [109,115].

PCTS they have gained increasing attention to investigate the effects of IRI [132,133,134]. As early as 1996, isolated hepatocytes and precision-cut-liver slices (PCLS) were compared to investigate the advantages and disadvantages in studying liver hypothermic preservation and reperfusion injury [133]. The utilization of PCTS as a model to study mechanisms has been reported by further groups in the recent decade. Hart et al. demonstrated that PCLS are a suitable in vitro model to study the consequences of ischemia and reperfusion. Thereby, the effects of different during hypothermic machine perfusion were investigated. Subsequently, it was concluded, that with an O_2_ saturation of 21%, the cultivation medium provides the best preservation technique [135]. Precision cut lung slices were developed as a hybrid model consisting of an in vivo ischemia period, followed by an in vitro reoxygenation to mimic cardiac death in lung transplantation [136] and myocardial rat slices have been utilized for studying biochemical and inflammatory processes during cold storage and after reperfusion [137,138]. Recently, also precision-cut kidney slices (PCKS) were described as a model for reperfusion injury [132]. Porcine kidneys previously subjected to 30 min of warm ischemia were utilized and different conditions were tested to eventually produce PCKS that are viable for 72 h [132]. PCTS offer an opportunity to study protective agents like antioxidants or potential pharmacological treatments to target different IRI-related mechanisms. In contrast to MP, a significantly lower dose of drugs and agents is needed and treatment and concentration protocols can be evaluated simultaneously. Smail et al. applied precision cut lung slices to analyze the role of inflammation in IRI in the lung, further investigating the protective role of adenosine. Lymphocytes were shown to enhance the inflammatory response and histological lesions after 4 h of warm ischemia [134]. Moreover, Schisandrin B has been shown to exert a protective function in IRI of the myocardium when applied on myocardial rat slices. It reduced the oxidative response, attenuated Activating transcription factor 6 (ATF6) and PKR-like endoplasmic reticulum (ER) kinase (PERK) signaling, and decreased ER stress-induced apoptosis [139].

### 4.3. Organoids 

Organoids are defined as 3D tissue structures grown from stem cells, posing an additional in vitro method that facilitates the study of oxidative stress in IRI. These structures consist of tissue-specific cell types that self-organize by cell arrangement and spatially restricted lineage commitment. They are either generated from either pluripotent embryonic stem (ES) cells, their synthetic counterparts induced pluripotent stem (iPS) cells or organ-restricted adult stem (aSC) cells. Moreover, patient-to-patient variability (present in, e.g., PCTS) can be circumvented while providing realistic microanatomy with a biomimetic environment [110]. Organoids are of particular interest in reproducing pathophysiological conditions and in studying the complexity of cellular interactions in various fields including IRI [110,140,141,142]. For example, Kip et al. have exposed intestinal organoids to hypoxia and reoxygenation, applying organoids as in vitro model for IRI. Subsequently, mass-spectrometry-based proteomics were conducted and protein dynamics and specific molecular mechanisms of IRI investigated [143]. Atypical physiology, limited maturation and lack of high-fidelity cells are a major hurdle to be overcome in this model. Organoids cannot be compared with normal tissue, as they lack vasculature and a functional immune system. Especially the lack of vasculature results in uneven spatiotemporal distribution of nutrients and oxygen. Moreover, that, cell-to-cell, batch-to-batch, organoid-to-organoid (within the same batch) and region-to-region variability (within the same organoid) are major drawbacks [141,142].

### 4.4. Organ-on-a-Chip

Organ-on-a-chip technology is a comparably new in vitro organ model system. It is a microfluidic device capable of mimicking the physical and chemical environment and thus, allow the different cell types of the respective organs to grow in an in vitro-like environment. This allows for drug testing within the pathophysiological conditions. More advantages of this model are longer shelf life, better hemodynamic and biocompatibility profiles, higher gas permeability as well as chemical sensitivity. Moreover, organ-on-a-chip is a rather cheap in vitro method. However, flow control and cell-to-liquid ratio are to be explored better, as low laminar flows are accompanied with little mixing. Moreover, that, surface effects may dominate volume effects, having an impact on adsorption of certain components to the surface. The surface itself may degrade during longer cultivation periods, affecting viability of studied cells. Organs-on-a-chip has been shown useful in studying IRI in the context of MP, since temperature, oxygenation and shear stress can be modelled to a degree [111,144,145].

There are already some reports available using this model in the context of IRI research. A microfluidics-based model for renal injury due to hypoxia and reperfusion was recently reported by Chethikkattuveli Salih et al. They cultivated primary human renal proximal tubule epithelial cells and primary human endothelial cells on the apical and basal sides of a porous membrane exposing them to hypoxic conditions [146]. Nemcovsky et al. presented a microfluidic IRI model with human endothelial cells. They demonstrated a significant increase in the expression of the inflammatory surface receptors, E-selectin and Intercellular adhesion molecule 1 (ICAM-1), in response to hypoxia. After reperfusion, an increase in ICAM-1 levels was recorded [147]. However, in this model volumes are very limited causing the surface effect to dominate the volume effect. Accordingly, this may translate into poorer quality of analysis. In addition, the liquids in question may not mix properly due to laminar flow at the intersection of several liquids [148].

## 5. Conclusions

Dynamic organ preservation has increased the number of organs available for transplantation. Oxidative stress-induced IRI remains a key event during organ retrieval, preservation and reperfusion, and profound understanding of the underlying mechanisms, especially during MP, is still missing. Research in the field of transplantation has been widely focused on in vivo studies both, in humans and animals. Of all the various MP methods mentioned above, normothermic machine perfusion of whole organs best mimics the in vivo setting by providing near physiologic conditions. However, access to human organs for research is very limited and animal models should be kept to a minimum following the 3R principles. Additionally, complex logistics, and the need for large human and financial resources associated with MP experiments underscore the need for reductionist in vitro models, which are suitable to study particular early aspects of redox stress-associated damage and to pretest potential therapeutic interventions. Several in vitro models posing valuable alternatives are listed in this review. Depending on research subject, there is a suitable model that allows for studying particular features, conditions and/or treatments in parallel. Isolated mechanisms can be assessed in a controlled, planned manner and in the absence of systemic influences. More often models are combined, in order to first explore, e.g., therapeutic agents and later apply them in ex vivo MP studies [149]. Different culturing and tissue engineering approaches for in vitro models have been reported to study IRI in more detail. So far, only a limited number of studies take MP specific requirements into account, however technical feasibility to study oxidative stress in the context of IRI was already demonstrated. This reinforces the need for further research and development in this field. By further deciphering of the mechanisms, novel strategies to prevent and counteract oxidative stress could be developed which may help to increase the number of organs available for transplantation.

## Figures and Tables

**Table 1:** Potential biomarkers for oxidative stress.

Category	Biomarker	Examples	Analysed Material	Detection Methods	Reference
Endogenous Antioxidants	CAT	SCS vs. HMP of Human Kidneys	Perfusate	Enzymatic activity measurement	[45]
		Isolated perfused Rat Heart	Tissue	Enzymatic activity measurement	[46]
	SOD	SCS vs. HMP of Human Kidneys	Perfusate	Enzymatic activity measurement	[45]
		Patients with coronary artery by-pass grafting surgery	Serum	Enzymatic activity measurement	[47]
		Reperfusion of rat kidney	Tissue	Biodiagnostics assay kit	[48]
		Isolated perfused Rat Heart	Tissue	MTT Assay	[46]
	GPx	SCS vs. HMP of Human Kidneys	Perfusate	Enzymatic activity measurement	[45]
Lipid Peroxidation	MDA	SCS vs. HMP of Human Kidneys	Perfusate	HPLC, ELISA (MDA-586 kit)	[45]
		Reperfusion of rat kidney	Tissue	Biodiagnostics assay kit	[48]
		Isolated perfused Rat Heart	Tissue	Conjugated to TBARS – Absorbance at 535nm	[46]
		Langendorff-perfused rat hearts	Tissue	HPLC/UV-Vis	[49]
	TBARS	SCS vs. HMP of Human Kidneys	Perfusate	Fluorometric Assay	[45]
		Isolated perfused Rat Heart	Tissue	TBARS-Assay	[46]
	F2 Isoprotanes	Transplanted Human Kidney	Plasma	Radioimmunoassay	[50]
		Reperfusion of Porcine Liver	Plasma	LQ/MS/MS	[51]
Protein Oxidation	Nitrotyrosine	Human Donor Livers before vs. after Transplantation	Tissue	Western Blot Analysis; Immunohistochemical Localization	[52]
		Reperfusion of Mice Kidney	Tissue	Western Blot Analysis	[53]
	Protein Carbonyl	Transplanted Human Kidneys	Plasma	DNPH Method	[54]
		NMP Porcine Kidney	Plasma	HPLC, ELISA, Immunoassays	[55]
		Langendorff-perfused rat hearts	Tissue	HPLC/UV-Vis	[49]
Nucleic Acid Oxidation	8-oxoguanine	HMP vs. SCS of canine hearts	Tissue	IHC	[56]
	8-hydroxy-2′-deoxyguanosine	Transplanted Human Kidneys	Plasma	ELISA	[54]
		Patients with coronary artery by-pass grafting surgery	Serum	ELISA	[47]
		Normothermic hepatic Ischemia/Reperfusion Model of Rats	Plasma Tissue	HPLC, IHC	[57]

Abbreviations: CAT: catalase; DNPH: 2,4-Dinitrophenylhydrazine; ELISA: Enzyme-Linked Immunosorbent Assay; GPx: Glutathione peroxidase; HMP: Hypothermic Machine Perfusion; HPLC: High Pressure Liquid Chromatography; IHC: Immunohistochemistry; LQ: Liquid Chromatography; MDA: Malondialdehyde; MS: Mass Spectrometry; MTT: 3-(4,5-dimethylthiazol-2-yl)-2,5-diphenyltetrazolium bromide; NMP: Normothermic Machine Perfusion; SCS: Static Cold Storage; SOD: Superoxide Dismutase; TBARS: thiobarbituric acid substance

## Data Availability

Not applicable.

## Abbreviations

ALT	Alanine Transaminase
aSC	Adult Stem Cells
AST	Aspartate Aminotransferase
ATF6	Activating Transcription Factor 6
ATP	Adenosine Triphosphate
Brg1	Brahma-related gene-1
CAT	Catalase
DAMP	Damage-Associated Molecular Pattern
DC	Dentritic Cell
DNPH	2,4-Dinitrophenylhydrazine
ECD	Extended Criteria Donor
ELISA	Enzyme-linked Immunosorbent Assay
ER	Endoplasmativ Reticulum
ERK	Extracellular Signal-regulated Kinase
ES	Embryonic Stem Cells
EVLP	Ex vivo Lung Perfusion
FOX	Forkhead Transcription Factor O
GPx	Glutathione peroxidase
HMGB1	High Mobility Group protein B1
HMP	Hypothermic Machine Perfusion
HO-1	Isoform Hämoxygenase-1
HOPE	Hypothermic Oxygenated Machine Perfusion
HPLC	High Pressure Liquid Chromatography
HTK	Histidine-Tryptophan-Ketoglutarate
I HC	Immunohistochemistry
ICAM-1	Intercellular Adhesion Molecule 1
IGL-1	Institute George Lopez-1
IκB	Inhibitor of κB Kinases
iPSC	Induced Pluripotent Stem Cells
IRI	Ischemia Reperfusion Injury
JNK	c-Jun N-terminal kinase
Keap1	Kelch-like ECH associated protein1
LDH	Lactate-Dehyrogenase
LQ	Liquid Chromatography
MAPK	Mitochondrial-activated Protein Kinases
MDA	Malondialdehyde
MP	Machine Perfusion
MS	Mass Spectrometry
mTOR	Mammalian Target of Rapamycin
MTS	3-(4,5-dimethylthiazol-2-yl)-5-(3-carboxymethoxyphenyl)-2-(4-sulfophenyl)-2H-tetrazolium
MTT	3-(4,5-dimethylthiazol-2-yl)-2,5-diphenyltetrazolium Bromide
NAD+	β-nicotinamide Adenine Dinucleotide
NFκB	Nuclear Factor-κB
NK	Natural Killer Cell
NMP	Normothermic Machine Perfusion
Nrf2	Nuclear factor-erythroid-2 Related Factor 2
PCKS	Precision-Cut Kidney Slices
PCLS	Precision-Cut Liver Slices
PCTS	Precision-Cut Tissue Slices
PERK	PKR-like Endoplasmic Reticulum Kinase
PI3/Akt	Phosphatidylinositol 3-kinase/Protein kinase B
ROS	Reactive Oxygen Species
SCS	Static Cold Storage
SDH	Succinate Dehydrogenase
SNMP	Subnormothermic Machine Perfusion
SOD	Superoxide Dismutase
TBARS	Thiobarbituric Acid Substance
UW	University of Wisconsin
